# Euglycemic diabetic ketoacidosis of unknown Etiology in a type 2 diabetic patient

**DOI:** 10.1093/omcr/omaf057

**Published:** 2025-06-27

**Authors:** Katrina J Villegas, Laura Guerrero, Hala Moussa, Islam Rajab, Abraam Rezkalla, Dhruv Patel, Mohammed A Qarqash

**Affiliations:** Department of Internal Medicine, St. Joseph’s University Medical Center, Paterson, NJ; Department of Psychiatry, Bergen New Bridge Medical Center, Paramus, NJ; Department of Internal Medicine, St. Joseph’s University Medical Center, Paterson, NJ; Department of Internal Medicine, St. Joseph’s University Medical Center, Paterson, NJ; Department of Internal Medicine, St. Joseph’s University Medical Center, Paterson, NJ; Department of Internal Medicine, St. Joseph’s University Medical Center, Paterson, NJ; Department of Medicine, An-Najah National University, Nablus, Palestine

**Keywords:** Euglycemic diabetic ketoacidosis, atypical DKA, metabolic acidosis without hyperglycemia, insulin-dependent diabetes complications, SGLT2 inhibitor-independent DKA, chronic kidney disease

## Abstract

Euglycemic diabetic ketoacidosis is a rare form of DKA characterized by severe ketosis and metabolic acidosis without marked hyperglycemia. We report a 45-year-old female with insulin-dependent diabetes and chronic kidney disease, initially presenting with acute heart failure, who later developed euglycemic DKA. Despite near-normal glucose levels, high anion gap metabolic acidosis with significant ketones highlighted this crisis. Treatment included fluids, insulin with dextrose, and intensive monitoring. This case underscores the need for early recognition of euglycemic DKA, especially in complex patients, to improve outcomes.

## Introduction

Euglycemic DKA is a rare form of ketoacidosis, presenting with marked ketosis and metabolic acidosis but without significant hyperglycemia, very characteristic of classical DKA. This is a life-threatening emergency that could be easily overlooked because its presentation is not typical of classical DKA, since most cases are associated with only mildly increased or normal levels of glucose. Diagnosis is more difficult, especially in patients not on SGLT2 inhibitors a common etiology for this situation. However, Euglycemic DKA can develop without the use of medicines, it is more likely to occur when a patient has certain predisposing conditions, such as chronic kidney disease, anorexia, gastroparesis, fasting, a ketogenic diet, or an alcohol use problem, which can cause episodes of carbohydrate starvation and subsequent ketosis. Pregnancy, pancreatitis, abnormalities of glycogen storage, surgery, infection, cocaine toxicity, cirrhosis, and insulin pump use are other factors that might cause euglycemic DKA. More than 20% of postoperative instances for type 1 diabetes mellitus patients who had bariatric surgery result in DKA, and they may be particularly vulnerable to euglycemic DKA [1]. The increasing use of SGLT2 inhibitors in the management of type 2 diabetes has heightened the clinical awareness for euglycemic DKA, though this condition could occur independently of the medication. These include symptoms like nonspecific nausea, vomiting, abdominal pain, and shortness of breath that could easily be mis-attributed to other conditions. Laboratory studies often demonstrate high anion gap metabolic acidosis with significant ketones; however, glucose may appear normal or near-normal due to this response, obscuring the occurrence of a metabolic crisis from the clinician. Diagnosis and treatment must be instituted expeditiously to avoid serious complications. Management includes replenishing fluids, correcting electrolyte abnormalities, insulin replacement supplemented by glucose to avoid hypoglycemia. For patients with diabetes, especially, a high index of suspicion is warranted, even in the absence of severe hyperglycemia, to provide optimal outcomes in cases of unexplained metabolic acidosis [2].

## Case report

A 45-year-old Spanish-speaking female with a history of insulin-dependent diabetes mellitus, hypertension, and chronic kidney disease presented with progressive shortness of breath, generalized swelling, and fatigue over several weeks. Additional symptoms included loose stools, decreased urine output, and abdominal pain. She denied similar prior episodes, recent travel, or sick contacts and reported adherence to her medications, including insulin and lisinopril, without using SGLT2 inhibitors, she also denied a history of prolonged fasting, adherence to a ketogenic or low-carbohydrate diet, recent weight loss, alcohol use, or any recent surgical procedures that could contribute to ketone accumulation.

On admission, she had tachycardia (130 bpm) and hypertension (systolic BP in the 160 s). Labs revealed elevated BUN (48 mg/dl), creatinine (3.03 mg/dl), and BNP (1697 pg/ml). Physical exam showed 2+ bilateral pitting edema, abdominal distension, and bilateral lung crackles. Diagnosed with acute decompensated heart failure, she received intravenous furosemide with symptomatic improvement (Table 1). To further assess potential underlying metabolic disturbances, additional laboratory evaluations were conducted, including serum osmolality, lactate, and toxicology screening.

**Table 1:** Laboratory results indicating Euglycemic diabetic ketoacidosis in a type 2 diabetic patient.

Parameter	Result	Reference Range
Blood Urea Nitrogen (BUN)	48 mg/dl	7–20 mg/dl
Creatinine	3.03 mg/dl	0.6–1.3 mg/dl
B-type Natriuretic Peptide (BNP)	1697 pg/ml	<100 pg/ml
Heart Rate	130 bpm	60–100 bpm
Blood Pressure	Systolic in 160 s	<120/80 mmHg
Serum Bicarbonate (initial)	22 mmol/l	22–29 mmol/l
Serum Bicarbonate (repeat)	13 mmol/l	22–29 mmol/l
Anion Gap	22	3–11
Blood Glucose (consistent)	200 s mg/dl	70–130 mg/dl (fasting)
Serum Ketones	Large	Negative

By the third hospitalization day, she developed nausea, vomiting, diarrhea, and poor oral intake. Labs showed a drop in serum bicarbonate (22 to 13 mmol/l), an elevated anion gap (22), large serum ketones, and glucose levels in the 200 s, leading to a diagnosis of euglycemic DKA. She had no evident triggers, including SGLT2 inhibitors or recent illnesses.

To exclude other potential causes of metabolic acidosis, an extensive workup was performed, including tests for lactic acidosis (serum lactate), adrenal insufficiency (morning cortisol), and toxic ingestion (methanol, ethylene glycol, salicylates). All results were within normal limits.

Serial arterial blood gas analysis was conducted to closely monitor acid–base status and confirm resolution of metabolic acidosis. Transferred to the ICU, she was treated with an insulin drip, D5–0.45% NS with potassium chloride, and supportive care. Serial labs showed improvement in bicarbonate levels and anion gap closure. She transitioned to subcutaneous insulin and resumed her home Lantus dose with an oral diet before discharge.

## Discussion

Euglycemic DKA is one of those states considered very infrequent; there exists acidosis and ketosis with absent marked hyperglycemia and hence diagnosis and therapeutic establishment also very challenging. Since hyperglycemia is a key diagnostic feature of classic DKA, clinicians may initially overlook euDKA, delaying appropriate treatment and increasing the risk of complications. This is the case-a 45-year-old who has been experiencing diabetes, hypertension, and chronic kidney disease-emphasized how DKA diagnosis itself is pretty cumbersome and becomes absolutely knotty under conditions, such as congestive heart failure obscuring its face. Congestive heart failure can further complicate metabolic homeostasis by inducing volume depletion, exacerbating insulin resistance, and triggering counterregulatory stress responses that promote ketosis. As the recent literature, including Diabetes Care, emphasizes, one should consider euglycemic DKA in patients with high anion gap metabolic acidosis and ketonemia after excluding other causes [3]. In clinical practice, a stepwise approach is essential, ensuring the exclusion of alternative causes such as lactic acidosis, toxic ingestions, adrenal insufficiency, and starvation ketosis before confirming the diagnosis of euDKA.

Unlike classic DKA, which presents with normal or mildly elevated glucose levels, recognition of euglycemic DKA is considerably delayed. The chronic kidney disease probably contributed to an impaired ketone clearance in this patient, while prolonged fasting or poor oral intake might have triggered ketogenesis [4]. This highlights the importance of recognizing CKD as a key risk factor, as impaired renal clearance allows for ketone accumulation, exacerbating acidosis despite relatively normal glucose levels. The absence of SGLT2 inhibitors further extends the need for recognition of nontraditional triggers for this condition, as emphasized in recent reviews. Although SGLT2 inhibitors are a well-known cause of euDKA, cases independent of these medications require careful evaluation of alternative precipitating factors such as infection, stress hypermetabolism, and catabolic states. Nausea, vomiting, and fatigue are usual symptoms that can be associated with euglycemic DKA and could resemble other disorders thus making diagnosis difficult or even wrong [5, 6].

Non-specific symptoms often lead to misdiagnosis as gastroenteritis, sepsis, or primary cardiac conditions, delaying targeted management and increasing the risk of worsening metabolic derangement. The cornerstone of management includes timely fluid resuscitation with balanced crystalloids, insulin administration to inhibit ketogenesis, and glucose supplementation to prevent hypoglycemia, as was shown in EMCrit. Fluid therapy must be carefully titrated in patients with CHF, balancing volume resuscitation against the risk of fluid overload, which may precipitate pulmonary edema. Most patients need to be admitted to the ICU for close monitoring and individualized therapy [7, 8]. ICU admission allows for serial arterial blood gas monitoring, electrolyte replacement, and continuous insulin infusion, which are critical to stabilizing acid–base balance. In the eScholarship, it was noted that even though there is no significant difference in mortality rates between euglycemic and hyperglycemic DKA, the former has a higher risk for treatment-related hypoglycemia since, by definition, glucose levels are generally normal at presentation [9]. This necessitates meticulous glucose monitoring and co-administration of dextrose-containing fluids to prevent iatrogenic hypoglycemia during insulin therapy.

This case underlines clinical awareness of euglycemic DKA, especially in patients with complex health conditions, to ensure timely intervention and improved outcomes. Given its atypical presentation, euDKA should be considered in any diabetic patient with unexplained metabolic acidosis, even in the absence of significant hyperglycemia. This case adds to the growing body of evidence that underlines the need for a proactive and comprehensive approach in the management of atypical presentations of DKA. Further studies are needed to refine diagnostic algorithms for euDKA and establish standardized treatment protocols, particularly in patients with coexisting renal and cardiovascular comorbidities.
